# Antibacterial and Biofilm Modulating Potential of Ferulic Acid-Grafted Chitosan against Human Pathogenic Bacteria

**DOI:** 10.3390/ijms19082157

**Published:** 2018-07-24

**Authors:** Chakradhar Dasagrandhi, Seulki Park, Won-Kyo Jung, Young-Mog Kim

**Affiliations:** 1 Marine-Integrated Bionics Research Center, Pukyong National University, Busan 48513, Korea; dasagrandhichakradhar@gmail.com; 2 Department of Food Science and Technology, Pukyong National University, Busan 48513, Korea; moojuk87@gmail.com; 3 Department of Biomedical Engineering, Pukyong National University, Busan 48513, Korea; wkjung@pknu.ac.kr

**Keywords:** antibacterial agent, antibiofilm, ferulic acid grafted chitosan, human pathogenic bacteria

## Abstract

The emergence of more virulent forms of human pathogenic bacteria with multi-drug resistance is a serious global issue and requires alternative control strategies. The current study focused on investigating the antibacterial and antibiofilm potential of ferulic acid-grafted chitosan (CFA) against *Listeria monocytogenes* (LM), *Pseudomonas aeruginosa* (PA), and *Staphylococcus aureus* (SA). The result showed that CFA at 64 µg/mL concentration exhibits bactericidal action against LM and SA (>4 log reduction) and bacteriostatic action against PA (<2 log colony forming units/mL reduction) within 24 h of incubation. Further studies based on propidium iodide uptake assay, measurement of material released from the cell, and electron microscopic analysis revealed that the bactericidal action of CFA was due to altered membrane integrity and permeability. CFA dose dependently inhibited biofilm formation (52–89% range), metabolic activity (30.8–75.1% range) and eradicated mature biofilms, and reduced viability (71–82% range) of the test bacteria. Also, the swarming motility of LM was differentially affected at sub-minimum inhibitory concentration (MIC) concentrations of CFA. In the present study, the ability of CFA to kill and alter the virulence production in human pathogenic bacteria will offer insights into a new scope for the application of these biomaterials in healthcare to effectively treat bacterial infections.

## 1. Introduction

The rapid emergence of different drug-resistant microorganisms and their inefficient prevention have become a potential threat to human health. *Listeria monocytogenes* (LM) is an important food-borne pathogen causing enteric diseases such as meningitis and septicemia [1]. Furthermore the listeriosis caused by LM is hard to treat and leads to hospitalizations and deaths [2]. Similarly, *Pseudomonas aeruginosa* (PA) is an important etiological agent responsible for nosocomial infections, cystic fibrosis, and other acute as well as chronic complications. It is difficult to eradicate the biofilms formed by PA from mucosal surfaces or infection sites and various biomedical devices [3]. *Staphylococcus aureus* (SA) is another important disease-causing agent responsible for community- and hospital-acquired infections. Multi-drug resistance development and biofilm formation by *S. aureus* results in chronic infections and leading to complicate treatment options and outcomes [4]. As a result of antimicrobial resistance development among pathogens and lack of novel antimicrobial agents, there is a need for an alternative effective control strategy to treat the pathogenic bacterial infection. In account of this, several researches have investigated the effectiveness of various natural products as antimicrobials, however, with limited success in the clinical trials. Hence, there is a large demand for structurally diverse and biocompatible molecules as antimicrobials for clinical therapy. In the present study, the research was focused on evaluating the antimicrobial potential of naturally derived chitosan and its phenolic derivative against important human pathogenic bacteria such as LM, PA and SA.

Chitosan (CS) is a natural biopolymer with antimicrobial, wound-healing, hypotensive, antidiabetic, antifungal, and drug-delivering properties [5]. There is a growing interest in developing CS derivatives with various bioactive agents in order to improve its biological properties and therapeutic applications in a synergistic way. Previous report showed that CS grafted with phenolic acids can be used as antimicrobial, antioxidant, hepatoprotective, and enzyme inhibitory agent [6,7,8]. Furthermore, in our previous study, we have shown that food phenolics grafted CS (sinapic, cinnamic and ferulic acid) exhibited better antimicrobial activity against skin pathogens [9]. However, details on the antimicrobial action and virulence inhibitory action of phenolic-CS are lacking. Hence, the current study was undertaken to study the mechanism of antibacterial action of ferulic acid-grafted chitosan (CFA) against human pathogenic bacteria and also its inhibitory effects on biofilm formation, biofilm eradication and also anti-virulence properties.

## 2. Results

### 2.1. Determination of Minimum Inhibitory Concentration (MIC) of Ferulic Acid-Grafted Chitosan (CFA)

Initially we performed the antimicrobial activity of unmodified chitosan (UMC) and their derivatives using the tryptic soy broth (TSB) growth media. The result of antimicrobial activity of UMC and CFA against the bacteria in TSB growth media was higher with the values of 64–2048 µg/mL and 64–512 µg/mL (Table 1), respectively. These results surprised us and we observed the influence of TSB growth media on the antimicrobial activity of UMC and CFA. To minimize the effect of growth medium, Muller–Hinton broth (MHB) medium was used for the antimicrobial study of UMC and CFA against the pathogenic bacteria. The antimicrobial results showed that both UMC and CFA exhibits a lower minimum inhibitory concentration (MIC) value of 64–128 µg/mL and 64 µg/mL, respectively.

### 2.2. Time-Dependent Growth Inhibition Studies

**Figure 1:**
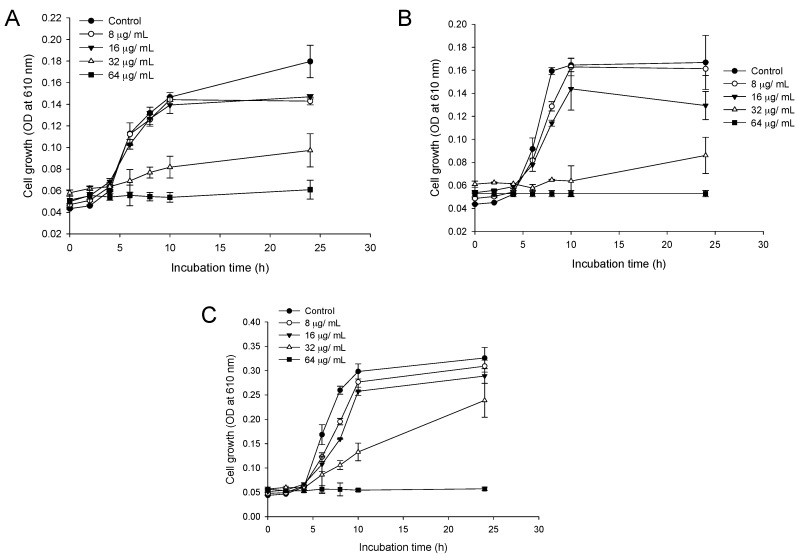
Time-dependent growth kinetics of different bacterial strains in the presence of ferulic acid grafted chitosan (CFA). (**A**) *Listeria monocytogenes* KCTC 3569; (**B**) *Pseudomonas aeruginosa* KCCM 11321; and (**C**) *Staphylococcus aureus* KCTC 1916 in the presence of MIC and sub-MIC concentrations of CFA. Mean cell growth ± standard deviation (SD) values of two independent experiments with duplicate values were presented (*n* = 4).

The time-dependent action of CFA on the growth of LM, PA and SA strains in MHB media was determined. From Figure 1A–C it is evident that CFA at 1× MIC (64 µg/mL) completely inhibits the growth of LM, PA and SA with no recurrent growth until 24 h. When the sub-MIC of CFA (32 µg/mL) was used, then there was decrease in lag phase with 4 h, however, with increase in incubation the growth was increased but there was a lower growth rate compared to the untreated control. Furthermore, at lower MIC values of CFA (4 and 8 µg/mL) we did not observe any profound changes in the growth pattern of test pathogens as compared to untreated controls. 

### 2.3. Antibacterial Activity of CFA

#### 2.3.1. Antibacterial Activity of CFA against Gram-Positive Bacteria 

**Figure 2:**
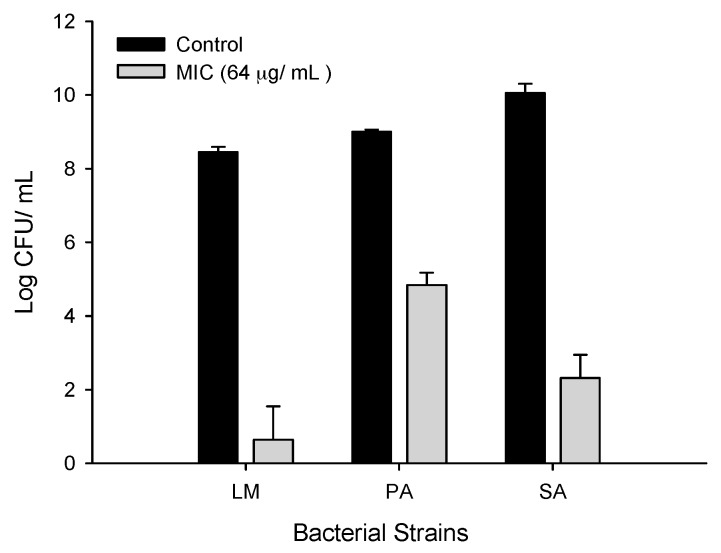
Determination of bacterial cell viability in presence of ferulic acid grafted chitosan (CFA). *Listeria monocytogenes* (LM) KCTC 3569, *Pseudomonas aeruginosa* (PA) KCCM 11321, and *Staphylococcus aureus* (SA) KCTC 1916 cells exposed to CFA at 1× MIC (64 µg/mL) concentration. Viabilities of CFA treated cells were compared with solvent (1% acetic acid) treated controls. Mean viability ± standard deviation (SD) values were presented (*n* = 3).

In order to investigate the antimicrobial action of CFA, viability assays were performed. To evaluate the viability, the bacterial cell culture with 5 × 10^5^ CFU/mL inoculum was used. The viability counts of test pathogens in the presence of CFA were represented in Figure 2. After 24 h incubation, the viability count of LM in an untreated control reached 8.6 log CFU/mL, whereas in the presence of CFA, a significant reduction in viable count up to 4.6 log reduction (99.9% inhibition) was observed (Figure 2). Similarly, in the case of SA treated with CFA, after 224 h incubation a similar reduction in viable count with the value of 3.2 log (99.9%) was observed. Interestingly, in the case of PA, CFA exhibited only 1.8 log reduction in viable counts as compared to the initial inoculum levels, which suggested a bacteriostatic action. The above results suggested that CFA has bactericidal action against Gram-positive pathogenic bacteria. 

#### 2.3.2. Effect of CFA on Bacterial Membrane Integrity 

**Figure 3:**
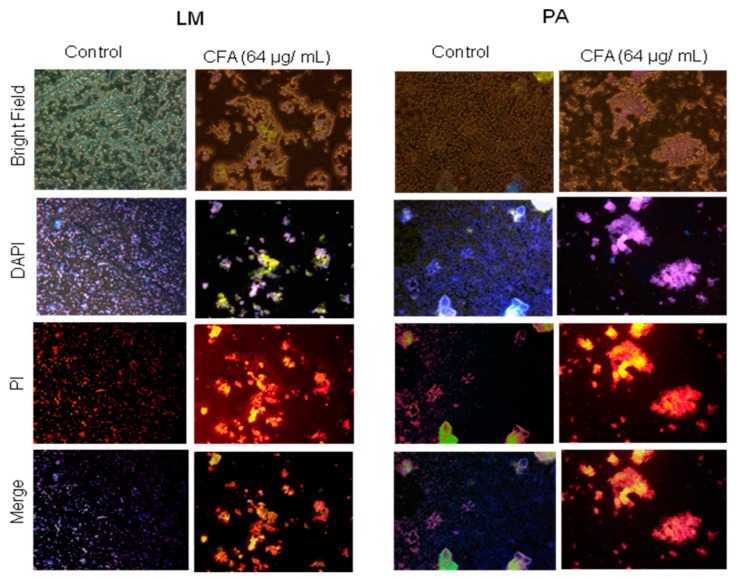
Fluorescence microscopic visualization of membrane integrity of *Listeria monocytogenes* KCTC 3569 (**A**) and *Pseudomonas aeruginosa* KCCM 11321 (**B**) either untreated (control) or exposed to ferulic acid grafted chitosan (CFA) at 1× MIC (64 µg/mL). Each experiment was performed three times and a representative image was presented. LM, *L. monocytogenes*; PA, *P. aeruginosa*; PI, propidium iodide; DAPI, 4′,6-diamidino-2-phenylindole.

To investigate the effect of CFA on bacterial cell membrane integrity, the cell culture of LM and PA treated with CFA were analyzed using confocal laser scanning microscopy (CLSM). In the cases of LM and PA (Figure 3), the merged images of propidium iodide (PI) and 4′,6-diamidino-2-phenylindole (DAPI) suggested that the untreated cells exhibited distinct blue color with relatively fewer cells exhibiting PI fluorescence. However, cells when treated with CFA (64 µg/mL) for 1 h caused a significant change in the colony morphology as the cells were found in aggregates (size, 10–500 µm) and contained 100–500 individual bacteria. The bacterial cells associated with these particles exhibited increased red fluorescence representing a possible membrane integrity and/or permeability change.

#### 2.3.3. Effect of CFA on Membrane Permeability in Gram-Positive Bacteria

**Figure 4:**
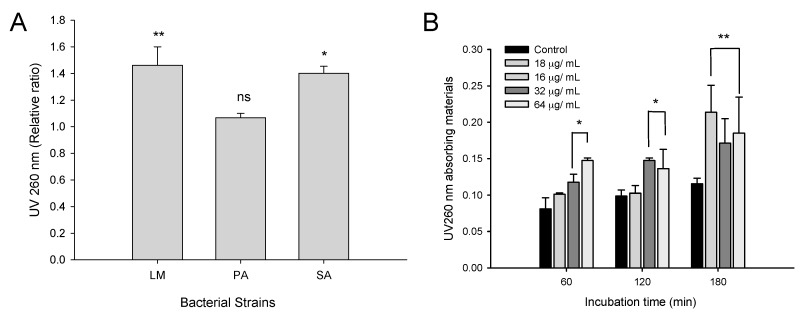
Determination of cellular material released from the cells (**A**) Absorbance (260 nm) value of the material released from *Listeria monocytogenes* (LM) KCTC 3569, *Pseudomonas aeruginosa* (PA) KCCM 11321, and *Staphylococcus aureus* (SA) KCTC 1916 exposed to CFA at 1× MIC (64 µg/mL) concentration; (**B**) Release of UV_260_ nm releasing material from the bacteria in presence of CFA (0–128 µg/mL) in a time-dependent manner. The leakage of materials was determined by measuring the absorbance at 260 nm using ultraviolet (UV)–visible spectrophotometer. The results were expressed as ratio of OD_260_ of CFA treated verses the OD_260_ of untreated controls. Mean ± SD values of two independent experiment values with triplicate values (*n* = 6) was presented. * *p* < 0.05, ** *p* < 0.01. ^ns^ Not significant.

Bacterial membrane permeability was monitored by quantifying the absorbance (at 260 nm) of material released from the cell with the help of an ultraviolet (UV)–visible spectrophotometer. The concentration of CFA for the study of material released from the cell was 1× MIC (64 µg/mL). Among the tested bacterial strains, in the case of LM and SA there was statistically significant (*p* < 0.05) release of materials with absorbance value of 1.53 ± 0.15 and 1.28 ± 0.12, respectively (Figure 4A). However, in the case of PA, there was no increase in the absorbance. Further results showed that CFA is permeabilized in a dose-dependent manner (Figure 4B). CFA at 0.25, 0.5 and 1× MIC (16, 32 and 64 µg/mL, respectively) caused a significant loss (*p* < 0.01) of UV_260_ nm absorbing material after 180 min compared to controls. At lower concentration (16 µg/mL) of CFA, there was negligible impact on the membrane permeability in LM within 120 min.

#### 2.3.4. Effect of CFA on the Cellular Morphology

**Figure 5:**
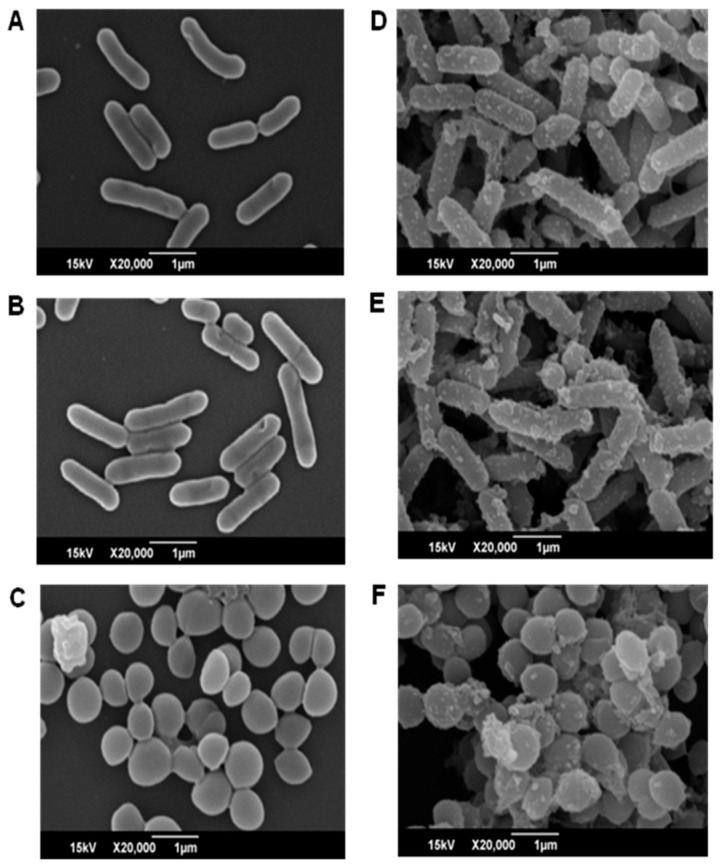
Scanning electron microscopy (SEM) analysis of the cells treated with ferulic acid grafted chitosan (CFA) at 1× MIC (64 µg/mL) concentration after 60 min. (**A**) *Listeria monocytogenes* KCTC 3569 (untreated); (**B**) *Pseudomonas aeruginosa* KCCM 11321(untreated); (**C**) *Staphylococcus aureus* KCTC 1916 (untreated); (**D**) *L. monocytogenes* KCTC 3569 (treated with CFA); (**E**) *P. aeruginosa* KCCM 11321 (treated with CFA); and (**F**) *S. aureus* KCTC 1916 (treated with CFA).

Scanning electron microscopy (SEM) analysis of bacteria treated with CFA at 1× MIC (64 μg/mL) concentration was carried out to observe any morphological changes induced by CFA in the test bacteria. The untreated cells of LM, PA, and SA exhibited normal cell shape with intact cell membrane integrity (Figure 5A–C). However, bacterial cells treated with CFA at 1× MIC level exhibited profound changes in the cell morphology and cells largely clustered together to appear in aggregates with gross increase in cell size when compared to their corresponding untreated controls (Figure 5D,E). Additionally, various CFA deposits are seen on the cell surface of the bacteria resulting in altered membrane integrity (Figure 5D,E). This present study suggested that the damage of bacterial membrane is induced by CFA which may be responsible for its antimicrobial action against the human pathogens.

### 2.4. Biofilm Inhibition Properties of CFA

**Figure 6:**
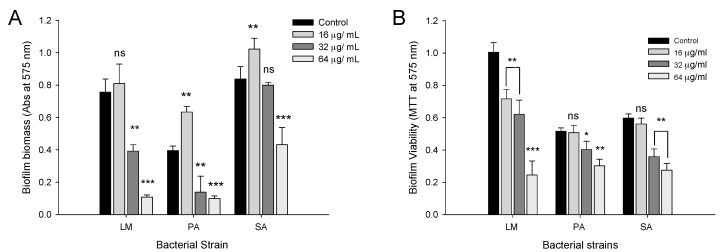
Time effect of ferulic acid grafted chitosan (CFA) on the biofilm formation by the bacteria. The biofilm formation was quantified by crystal violet staining method (**A**) and metabolic activity determined by MTT assay (**B**). Mean values of three replicates (±SD) are represented. LM, *Listeria monocytogenes* KCTC 3569; PA, *Pseudomonas aeruginosa* KCCM 11321; SA, *Staphylococcus aureus* KCTC 1916. * *p* < 0.05, ** *p* < 0.01, *** *p* < 0.001. ^ns^ Not significant.

The ability of CFA to prevent the adhesion of bacterial biofilm was investigated by the crystal violet staining method. From the results it is evident that CFA showed a dose-dependent inhibition in the biofilm attachment to the polystyrene surface. The result obtained from, the crystal violet staining indicates that CFA inhibits bacterial cell adhesion to the surface in a dose-dependent manner (Figure 6A). CFA at 1× MIC (64 µg/mL) concentration significantly (*p* < 0.001) reduced the biofilm formation of LM, PA, and SA with the inhibition values of 89.1 ± 7.5%, 75 ± 2.7% and 52.5 ± 3.5%, respectively. Similarly, CFA at 0.5× MIC (32 µg/mL) also caused a significant (*p* < 0.01) inhibition of LM, PA and SA biofilms by 48.1 ± 2.1%, 24.5 ± 13.9% and 25 ± 3.7%, respectively. Interestingly CFA at 0.25 MIC (16 µg/mL) resulted in an enhanced biofilm formation as evidenced from a crystal violet staining assay (Figure 6A). From MTT assay results, it is evident that 0.25 MIC of CFA had no profound effect on the metabolic activity of all test strains as compared to the controls (Figure 6B). However, increasing the concentration of CFA up to (1× MIC) also resulted in significant (*p* < 0.01) loss of metabolic activity of LM, PA, and SA biofilms by 75.1 ± 9.5%, 30.8 ± 5.3%, and 54.0 ± 4.7%, respectively (Figure 6B), which are supporting the results as obtained in biofilm inhibition experiments.

### 2.5. Disruption of Preformed Mature Biofilms by CFA

**Figure 7:**
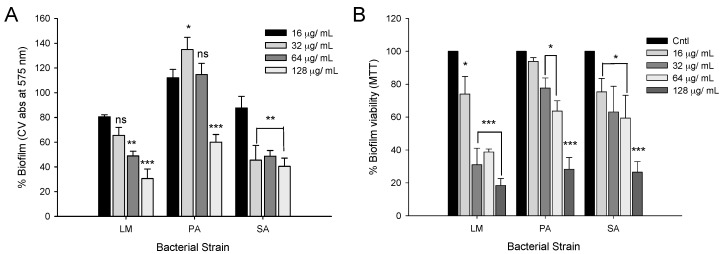
Disruption effect of ferulic acid grafted chitosan (CFA) on the preformed mature biofilms of bacteria. Disruption of mature biofilms of *Listeria monocytogenes* KCTC 3569 (LM), *Pseudomonas aeruginosa* KCCM 11321 (PA), and *Staphylococcus aureus* KCTC 1916 (SA) in presence of CFA (16–128 µg/mL) as quantified by crystal violet staining method (**A**) and metabolic activity determined by MTT method (**B**). Mean values of three replicates (±SD) was represented. * *p* < 0.05, ** *p* < 0.01, *** *p* < 0.001. ^ns^ Not significant.

Results obtained from the disruption study of preformed mature biofilm by CFA showed a dose-dependent clearance of biofilm biomass (Figure 7A). Significant dispersion (*p* < 0.001) of biofilm biomass by CFA at 2× MIC concentration was observed in LM, PA and SA with the values of 69.6%, 40.1% and 59.5%, respectively (Figure 7A). Similarly, CFA also exhibited a dose-dependent reduction in the viability of mature biofilms (Figure 7B). At 2× MIC concentration of CFA the large proportion of biofilm cells in case of LM, PA and SA with values of 82%, 71% and 73.5%, respectively, were found to be metabolically inactive (*p* < 0.001). 

**Figure 8:**
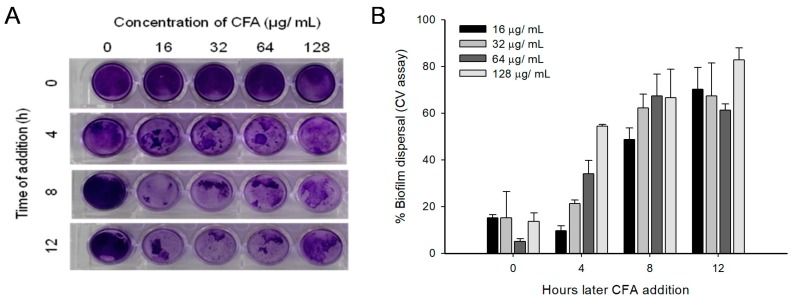
Effect of ferulic acid grafted chitosan on the dispersal of mature biofilm of *Listeria monocytogenes* (LM) KCTC 3569 after adding CFA at different time interval. (**A**) Photograph of a plate showing LM biofilms eradication from the surface of polystyrene and (**B**) Estimation of biofilm remaining on the microtiter plates in presence of CFA at different time periods. Mean biofilm ±SD values of three replicates is represented.

Furthermore from the time-dependent addition of the CFA experiment, a significant amount of mature biofilm was found to be detached from the surface when CFA was added after 4 h (Figure 8A,B). Experiments were conducted to test whether CFA can also disrupt the biofilms formed by polymicrobial or mixed biofilms. 

**Figure 9:**
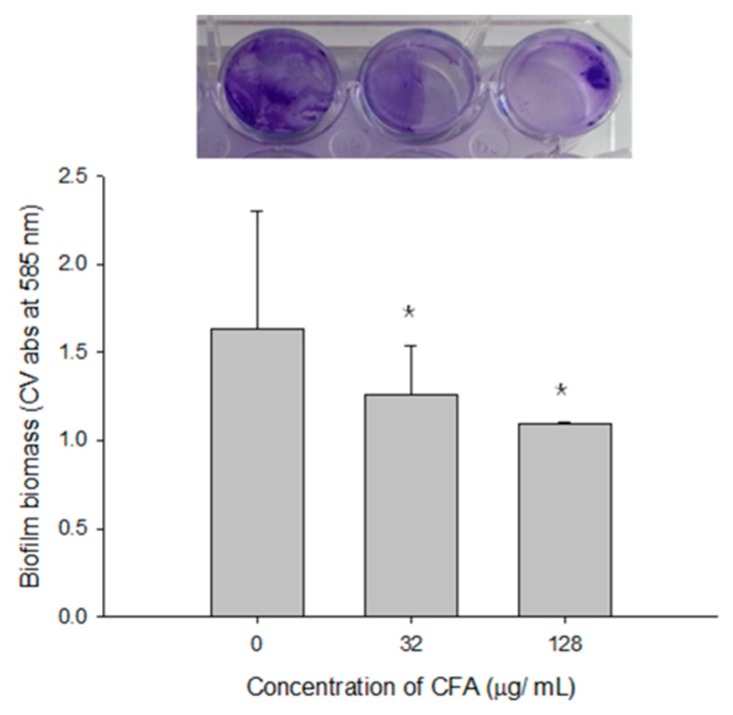
Inhibitory effect of ferulic acid grafted chitosan (CFA) on the polymicrobial biofilms formed by *Listeria monocytogenes* KCTC 3569, *Pseudomonas aeruginosa* KCCM 11321, and *Staphylococcus aureus* KCTC 1916. Mature biofilms of 3 strains were incubated with CFA (32 or 128 µg/mL) for 24 h. The biofilm biomass was compared with the acetic acid treated controls. Mean ±SD of triplicate values are represented. * *p* < 0.05.

It is also evident that CFA at both sub-MIC (32 µg/mL) and supra-MIC (128 µg/mL) concentration could effectively (*p* < 0.05) disperse the mature polymicrobial biofilms composed of LM, PA, and SA as established on a polystyrene surface (Figure 9).

### 2.6. Effect of CFA on Bacterial Motility

**Figure 10:**
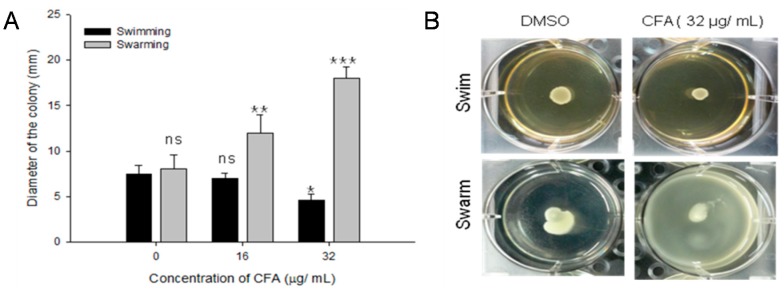
Effect of ferulic acid grafted chitosan (CFA) on swarming and swimming motility of *Listeria monocytogenes* KCTC 3569. (**A**) Swarming and swimming motility of *L. monocytogenes* grown in the presence of 1% acetic acid (control) or CFA with concentration of 16 µg/mL and 32 µg/mL; (**B**) A pictorial image of the plate showing the swarming and swimming motility of *L. monocytogenes* in the presence or absence of CFA.

In swimming motility assays, CFA incorporated into test agar plates showed a significant reduction in swimming motility of the test bacterial strain (LM) (Figure 10A). In the absence of CFA the swimming halo size of LM was observed to be 7.4 ± 0.9 mm after 24 h of incubation, however, in the presence of CFA at 0.5 MIC (32 µg/mL) concentration the motility halo size was significantly (*p* < 0.05) reduced (Figure 10A). However, CFA at 0.25 MIC (16 µg/mL) concentration could not show any inhibitory effect on LM swimming motility. In contrast to the swimming motility assay, CFA exhibited a dose-dependent stimulating effect on the swarming motility by LM. In the presence of CFA at 32 µg/mL concentration, a statistically significant (*p* < 0.001) increased size of swarming motility halo (18.5 ± 1.2 mm) was observed as compared to the controls (9.3 ± 1.6 mm). Figure 10B represent the image of the plate showing the swarming and swimming motility of *L. monocytogenes* KCTC 3569 in the presence or absence of CFA.

## 3. Discussion

The ability of food-borne pathogenic bacteria to develop resistance against conventional antibiotics resulted in an increased mortality rate due to listeriosis and other pathogenic bacterial infections, which has drawn attention to the urgent need to find novel control strategies. Hence, the current study was focused on developing an antipathogenic agent that could effectively inhibit pathogens like LM, PA, and SA by exerting a selection pressure over the growth. Recent and past studies showed that the phenolic acids used as preservatives in food applications due to their antioxidant property [10]. Ferulic acid is one example of a phenolic acid reported to exhibit antimicrobial potential with less cytotoxicity and there are several more biological activities [11]. In order to further extend the application of ferulic acid with a broad range of action and high antimicrobial efficiency, the conjugation of this agent with biocompatible and biodegradable polymers is considered the effective approach. Thus the present study was aimed at exploring the antibacterial and antibiofilm properties of conjugated ferulic acid with a naturally derived polymer i.e., chitosan against food-borne pathogenic bacteria such as LM, PA and SA. 

MIC determination revealed the potent in vitro antibacterial propensity of CFA (MIC, 64 μg/mL) against all three tested pathogens. Here we show that, CFA could inhibit the food-borne pathogens effectively with MIC (64 µg/mL) against Gram-positive and Gram-negative test pathogens. The above results are in close agreement with the reports of gallic acid grafted chitosan with the MIC value of 16–64 µg/mL against the Gram-positive bacteria, however, against the Gram-negative bacteria gallic acid grafted chitosan showed higher MIC value of 128–512 µg/mL [12]. Similarly, ferulic acid grafted chitosan was also reported to exhibit antimicrobial activity with an MIC range of 16–256 µg/mL against acne-causing *P. aeruginosa* and *S. aureus* strains [9]. Earlier reports showed that gallic acid grafted chitosan exhibits delayed response in 10 h to cause bactericidal action (>5 log reduction) against *Escherichia coli* and *S. aureus* strains [12]. In contrast to the above reports, our results showed that CFA caused a rapid bactericidal action (>4 log CFU/mL reduction) against the Gram-positive strains and bacteriostatic action (1.8 log reduction) against Gram-negative bacteria such as PA. 

The antibacterial activity of CFA observed in this study is expected to be due to its membrane-targeting action. Cells treated with CFA exhibited greater loss of membrane permeability due to membrane integrity loss induced by CFA interaction with the bacterial cells. PI is cell impermeable which selectively binds the nuclear material of membrane integrity compromised bacterial cells. Likewise, image analysis using fluorescence microscopy revealed that CFA treated LM and PA cells rapidly uptake PI. Furthermore, CFA at MIC concentration caused the release of cytoplasmic materials from Gram-positive bacterial cells such as LM and SA in a bacteriolytic mode of action. The above results are in close agreement with those previously reported for other phenolic acid-grafted chitosan against Gram-positive bacteria and the mode of action was in a dose-dependent manner by damaging the cell membrane [12]. Surprisingly, CFA at 1× MIC concentration did not cause bacteriolysis in case of PA and these results are in contrast to the membrane permeability induced by gallic acid-grafted chitosan in *E. coli* [12]. The membrane integrity and change in cell morphology were further supported by SEM results which showed that majority of the CFA-treated cells increased in size with irregular morphology and altered membrane integrity. Earlier, it was reported that interaction of positively charged chitosan with the negatively charged cell membrane of bacteria causes bactericidal action [13]. However, the better bactericidal activity of CFA is observed in our study due to the synergistic antimicrobial action of ferulic acid and chitosan on multiple cellular targets in bacteria. Ferulic acid, being hydrophobic in nature is able to quickly partition the lipid bilayer and alter membrane permeability. Additionally, the cell-binding action of chitosan alters the structure and function of cell surface appendages like teichoic and lipoteichoic acids which result in weak cell wall assembly and internal cell membrane permeability and lysis [14]. 

Bacterial adhesion is a complex process which is affected by physico-chemical factors, material surface properties, environmental factors, and biological properties such as the presence of fimbriae, flagella, and the production of extracellular polymeric substances [15,16]. Therefore, prevention of bacterial adhesion could prevent biofilm formation and its virulence production. In this study, CFA at 1× MIC and 0.5× MIC (64 and 32 µg/mL, respectively) concentrations prominently inhibited the biofilm formation and its metabolic activity by all test pathogens. In our opinion, the biofilm preventing role of CFA could be due to its sub-lethal effects on the viability. Earlier it was reported that biofilm prevention activity of gallic acid and ferulic against PA, LM, and SA biofilms was high at >1 mg/mL [11]. Similarly, it is reported that chitosan (2.6 kDa) at >1 mg/mL prevents biofilms formation of LM, PA, and SA [17]. Also, alginate, a carboxyl methyl derivative of chitosan with varying degrees of efficiency was reported to inhibit biofilm formation in pathogens [18]. However, the antibiofilm effect of CFA observed in our study is more effective compared to the antibioiflm property reported for catechol derivatives of chitosan against *S. epidermidis* biofilms [19]. 

Biofilm formation is a multi-step process and cells in mature biofilms are associated with extracellular polymeric substances (EPS) composed of extracellular polysaccharide, eDNA, proteins and several other biofilm materials making it resistant to various harsh environmental factors like salinity, antimicrobials and disinfectants [20]. The current study shows that CFA at sub- and supra-MIC levels was effective in reducing the biofilm quantity and metabolic activity (range, 50–70%) of LM, PA, and SA. Previous reports showed that individual phenolic acids like gallic acid and ferulic acid can also inhibit the biofilm (>50%) and metabolic activity (>70%) of *E. coli*, PA and LM [11]. Chitosan has a capability to eradicate mature biofilms of bacteria and its penetration into the biofilm matrix depended on its size, charge, deacetylation degree, and interaction with anionic exopolymeric matrix [21]. The exact mechanism of antibiofilm action of CFA is not clear. However, based on the present study, we speculated that biofilm eradication by CFA can be a multi-step process in which ferulic acid disassembles the biofilm exo-polymeric matrix and facilitates chitosan’s penetration and hence its interaction with the cell surface and cell membrane components, which affects multiple cellular targets like adhesion proteins, cell surface proteins, exopolymers, and also interference in cell-to-cell communication of mature biofilms. 

Bacterial motility is an important factor responsible for the biofilm spread, adhesion, and maturation processes. In this study, the sub-MIC (32 µg/mL) concentration of CFA not only inhibited mature biofilms but also dose-dependently altered bacterial motility against LM. CFA reduced the swimming motility of LM but positively affected the swarming motility. The increase in swarming motility by CFA can be interpreted through the findings of Caiazza et al. [22], who reported a close link between swarming motility and biofilm formation in PA by flagellar reversals and production of pel polysaccharides. 

Phenolic acid-functionalized chitosan has been shown to possess antioxidant, antitumor, antimicrobial, antidiabetic properties and also used as adsorptive, encapsulation, and packing materials for immobilization and delivering of drugs [23,24,25,26,27]. In addition to the above findings, the present study evaluates the antimicrobial and virulence inhibitory potential of CFA against human pathogenic bacteria. CFA effectively inhibited multiple pathogens by altering membrane integrity and cell permeability characteristics. The ability of CFA to inhibit human pathogenic bacteria with the ability to prevent initial biofilm formation as well as disrupting the mature biofilms represents a new possibility of application of CFA in food, healthcare and the effective control of human pathogenic bacterial infections. CFA, being a non-toxic antioxidant agent, can improve oxidative stability and control pathogenicity by inhibiting the production of virulence factors and hence can be effective in the protection of food. Thus, a more detailed study on the application of CFA in a food model system is required.

## 4. Materials and Methods

### 4.1. Chemicals and Reagents

Medium molecular weight chitosan (310 kDa in size with 85% degree of deacetylation) was obtained from Kitto Life Co. (Seoul, Korea). Ferulic acid, DMSO, EtBr, MTT, PI, and DAPI were obtained from Sigma-Aldrich (St. Louis, MO, USA). CFA was prepared according to the previously described method [6]. 

### 4.2. Bacterial Strains and Culture Conditions

*P. aeruginosa* KCCM 11321 (PA) was obtained from the Korean Culture Center of Microorganisms (Seoul, Korea). *L. monocytogenes* KCTC 3569 (LM) and *S. aureus* KCTC 1916 (SA) were from the Korean Collection for Type Cultures (Daejeon, Korea). Bacterial stocks were prepared in TSB (Difico, Detroit, MI, USA) with 30% glycerol and stored at −80 °C for future work. Prior to each experiment, a loop full culture was used to inoculate TSB broth and incubated at 32 °C for 24 h. MHB (Difico) supplemented with 1% glucose was used for LM biofilm experiments. Similarly, TSB supplemented with 1% glucose was used for PA and SA biofilm assays. Overnight cultures diluted to 100-fold in the respective media to OD of 0.4 at 610 nm (10^8^ CFU/mL) were used for subsequent assays.

### 4.3. Antibacterial Assay

The MIC was determined through micro-dilution method as described by clinical and laboratory standards institute (CLSI) [28]. All the samples were initially dissolved in 1% acetic acid and diluted in a 96 well microtitre plate containing MHB/TSB (0.1 mL) to obtain a final concentration of CFA ranging from 128 to 2 µg/mL. The mid-log phase bacteria were diluted to 10-fold in MHB (5 × 10^5^ CFU/mL) and 0.1 mL aliquots were added to the wells and incubated at 37 °C for 24 h. The optical density of the cell culture was determined using a microplate reader at a wavelength of 610 nm (Multiskan GO, Thermo Fisher Scientific Korea Ltd., Seoul, Korea). The lowest concentration of CFA which inhibited the visible growth of bacteria was considered the MIC value. Minimum bactericidal concentration (MBC) was determined by spreading 100 µL of the bacterial cultures from MIC assay on MH-agar plates and incubated at 32 °C for 24 h. The lowest concentration that allowed the formation of <5 colonies on each plate was considered as the MBC.

### 4.4. Growth Curves and Viability Assays

The time-dependent growth inhibitory effect of CFA against test pathogens was measured using broth microdilution method. From overnight cultures, inoculums (5 × 10^5^ CFU/mL) was prepared and incubated with MHB containing various concentration of CFA (0–128 µg/mL) and incubated at 32 °C for 24 h. The optical density (OD_610_ nm) of bacterial cell was measured once in every 2 h by multi-label plate reader (Thomson Scientific, Philadelphia, PA, USA). Additionally, the bacterial cell viability in presence of 1× MIC concentration of CFA was determined according to the method as described previously [29] with a slight modifications. One mL of test bacteria (5 × 10^5^ CFU/mL) was added to 19 mL of MHB with CFA or without CFA (untreated control). Following 24 h incubation, samples were taken and viable counts were determined by serially diluting in sterile 0.1 M phosphate buffer saline (PBS). Aliquot (0.1 mL) of serial dilution sample was placed on to tryptic soy agar (TSA, Difico, Detroit, MI, USA) plates, colonies were counted after 24 h incubation at 35 °C.

### 4.5. Quantification of Cytoplasmic Materials Released from the Cell

The bacteriolytic action of CFA was determined by quantifying the release of cytoplasmic materials using UV–visible absorbance spectrophotometer at wavelength of 260 nm according to the method as described previously [30] with slight modification. Briefly, overnight test bacteria were diluted at 1:100 in MHB and further grown till OD_610_ of 0.5 is reached. The cells were harvested (5000× *g* for 3 min) and re-suspended in 0.1 M PBS (0.5 OD) and incubated with the test bacterial strain at 1× MIC (64 µg/mL) for 4 h. At every 30 min time intervals, aliquot of 1 mL was withdrawn and centrifuged (5000× *g* for 3 min) and the supernatant was filter-sterilized with membrane filter of pore size 0.2 µm. The absorbance of clear supernatant was quantified at 260 nm. Solvent (1% acetic acid alone) or untreated samples were used as negative and blank controls in this study. The results were given as ratio release (CFA/control). 

### 4.6. P*ropidium Iodide* (PI) and 4′,6-Diamidino-2-Phenylindole (DAPI) Uptake Assay

To check the bacterial membrane damage caused by CFA in LA and PA, fluorescence microscopic analysis was conducted using fluorescent indicators such as, PI and DAPI. DAPI generally stains all cells in a population, whereas PI penetrates only when the cell membrane integrity is compromised causing a reduction in DAPI fluorescence in the presence of both dyes. Briefly, LM and PA cells (0.5 OD) in 0.1 M PBS were treated with CFA (64 µg/mL) for 60 min and the cells were harvested by centrifugation (5000× *g* for 3 min). The harvested cells were re-suspended in 0.1 M PBS, incubated with DAPI (5 µM) and PI (5 µM) for 5 min, and excess stain was removed by centrifugation (5000× *g* for 3 min). The collected cells were fixed on glass slides and observed under confocal laser scanning microscope (CKX53, Olympus Inc., Tokyo, Japan) equipped with 40× objective lenses, using the red channel (λ_ex_ 504 and λ_em_ 540) and the blue channel (λ_ex_ 358 and λ_em_ 461).

### 4.7. Scanning Electron Microscopy (SEM)

The morphology of test bacteria treated with CFA was determined using SEM according to the method as described previously [31]. Briefly, bacterial cells (5 × 10^5^ CFU/mL) were incubated in presence of 64 µg/mL (MIC) of CFA for 1 h. Aliquot (10 µL) sample was placed on cover slips and air dried at 28 °C for 30 min. Later the samples were fixed using 2.5% glutaraldehyde solution (0.1 M PBS, pH 7.2) at 4 °C for 4 h. Fixed samples were then dehydrated in increasing concentrations of ethanol (10%, 25%, 50%, 75%, 95% and 100%) and isoamyl alcohol (100%) for 10 min. The cover slips were finally air dried, mounted and then sputter coated with gold-palladium and analyzed under a scanning electron microscope (S-3400N, Hitachi, Tokyo, Japan). Bacterial cells treated with 0.1 M PBS (pH 7.4) was used as control.

### 4.8. Biofilm Modulating Potential of CFA

#### 4.8.1. Inhibition of Initial Attachment

The effect of CFA on biofilm formation by LM, PA and SA was tested in 24-well microtiter plates by modified crystal violet staining assay method as described by Jadhav et al. [32] with some modifications. Overnight grown cultures of test bacteria were 100-fold diluted to get 5 × 10^8^ CFU/mL. The biofilm of the test bacteria was formed by inoculating 0.1 mL aliquot from the culture medium in to the biofilm growth medium (1% supplemented MHB for LM and 1% glucose supplemented TSB for PA and SA) previously adjusted with CFA (MIC, 1/2 MIC, and 1/4 MIC). The microtiter plates were incubated under static conditions at 32 °C for 48 h. Subsequently, planktonic cells and the media were removed, the wells were rinsed three times with sterile 0.1 M PBS and the biofilm biomass estimated by crystal violet method as described below.

#### 4.8.2. Effect on Preformed Biofilms

An overnight culture of test bacteria was diluted to 100-fold in MHB to obtain an initial inoculum of 5 × 10^8^ CFU/mL and an aliquot (100 µL) was used to inoculate the 24 well microtiterplate containing 0.9 mL of the biofilm growth medium and incubated at 32 °C for 48 h to form mature biofilms. After incubation, the mature biofilms were washed with 0.1 M PBS twice to remove non-adherent bacterial cells and fresh biofilm growth media (1 mL) was added containing various concentrations of CFA (0–128 µg/mL) and further incubated at 32 °C for 48 h. At the end of incubation, the biofilms were processed and the biofilm biomass was quantified by crystal violet and MTT methods (described below). 

#### 4.8.3. Biofilm Estimation by Crystal Violet Staining Method

Following incubation of the test bacteria in presence of CFA, the plates containing planktonic cells were removed and loose adherent cells were washed with sterile distilled water three times. The plates were then air dried at 40 °C for 30 min. The cells in the biofilm were then stained with 1% crystal violet (100 µL) and incubated at 28 °C for 15 min and later washed three times with sterile distilled water. The crystal violet bound to biofilm was then extracted with 30% acetic acid (125 µL), an aliquot (100 µL) was from each well was transferred to a new plate, and the absorbance at 595 nm was determined (Multiskan GO, Thermo Fisher Scientific Korea Ltd., Seoul, Korea). The percentage biomass formation was determined using the following equation.

Percentage Biofilm Formation = [{Test Sample OD_595_ nm/Control sample OD_595_ nm} × 100].

#### 4.8.4. Biofilm Viability Determination by MTT Assay

The effect of CFA on the viability of bacterial strains was assessed by using the MTT assay [33]. The firm biofilms on microtier plates were incubated with 100 µL of MTT (10 mg/mL stock) and incubated for 3 h at 37 °C and the insoluble purple formazan formed in the plates was extracted with DMSO (100 µL), centrifuged (3000× *g*) for 5 min, and the clear supernatant was transferred to a new 96-well plate and the absorbance at 570 nm using the microplate reader (Multiskan GO, Thermo Fisher Scientific Korea Ltd., Seoul, Korea).

#### 4.8.5. Effect on Bacterial Motility

In order to test the inhibitory effect of CFA on mobility of LM and PA, a soft agar motility assay was performed according to the previously described method [34] with slight modification. Briefly, swim agar (10 g/L tryptone, 5 g/L NaCl, and 0.3% agar) and swam agar (8 g/L nutrient broth, 5 g/L glucose, 0.5% agar) plates were prepared by supplementing sub-MIC of CFA (32 µg/mL) and the plates devoid of CFA served as controls. An overnight culture of LM and PA was centrifuged at 3000× *g* for 5 min and washed three times with 0.1 M PBS. A total of 10 µL of re-suspended culture (~5 log CFU/mL) was stab inoculated at the center of the swim agar plate (swim assay) or point inoculated at the center of the swarm agar plate (swarm assay) and the plates were incubated at 28 °C for 24 h for the swimming and 72 h for swarming analysis.

#### 4.8.6. Statistical Analysis

Where applicable, the data were analyzed for statistical significance by a two tailed student’s *t* test for unpaired data using Graph Pad Prism’version 5.00 (GraphPad Software, San Diego, CA, USA).

## 5. Conclusions

In conclusion, we report the antimicrobial and biofilm modulating properties of the conjugated ferulic acid (CFA) against the food-borne Gram-positive and Gram-negative pathogenic bacteria. In the present study, CFA at varying concentration showed different activity to the different pathogenic bacteria. In the case of LM and SA, it shows bactericidal activity, whereas for PA it shows bacteriostatic activity at the MIC (64 µg/mL) concentration of CFA. The mode of antimicrobial action of CFA was found due to the cell membrane damaging activity as confirmed structurally and functionally. Furthermore, CFA also showed biofilm-inhibiting properties to LM, PA and SA in a dose-dependent manner. Not only does CFA inhibit biofilm formation, it also has properties to eradicate the preformed mature biofilms of these bacterial strains. In addition, the dual properties of CFA exhibiting both antibacterial and antibiofilm against pathogenic bacteria will open a new possibility for its application in different food industries and health care.

## Figures and Tables

**Table 1:** Determination of minimum inhibitory concentration (MIC) of unmodified chitosan (UMC) and ferulic acid-grafted chitosan (CFA) against representative test pathogens in different culture media.

Strains	Tryptic Soy Broth (TSB)		Muller-Hinton Broth (MHB)	
	UMC	CFA	UMC	CFA
*Listeria monocytogenes* KCTC 3569	64	64	64	64
*Staphylococcus aureus* KCTC 1916	2048	512	128	64
*Pseudomonas aeruginosa* KCCM 11321	1024	512	128	64

## Abbreviations

LM	*Listeria monocytogens*
PA	*Pseudomonas aeruginosa*
SA	*Staphylococcus aureus*
CS	Chitosan
UMC	Unmodified Chitosan
CFA	Chitosan Conjugated Ferulic Acid
MBC	Minimum Bactericidal Concentration
MIC	Minimum Inhibitory Concentration
PI	Propidium Iodide
DAPI	4′,6-Diamidino-2-phenylindole
SEM	Scanning Electron Microscopy
DMSO	Dimethyl Sulfoxide
MTT	3-(4,5-Dimethylthiazol-2-yl)-2,5-diphenyltetrazolium Bromide
PBS	Phosphate Buffered Saline
TSB	Tryptic Soy Broth
MHB	Muller-Hinton Broth
CLSM	Confocal Laser Scanning Microscopy
